# Recent developments on psychological factors in medically unexplained symptoms and somatoform disorders

**DOI:** 10.3389/fpubh.2022.1033203

**Published:** 2022-11-04

**Authors:** Ricarda Mewes

**Affiliations:** Faculty of Psychology, University of Vienna, Vienna, Austria

**Keywords:** psychological factors, medically unexplained symptoms, somatoform disorders, somatic symptom disorder, depression

## Abstract

Somatic symptoms which are not fully explained by a medical condition (medically unexplained symptoms) have a high relevance for the public health. They are very common both in the general population and in patients in health care, and may develop into chronic impairing conditions such as somatoform disorders. In recent years, the relevance of specific negative psychological factors for the diagnosis and the stability of somatoform disorders and for the impairment by medically unexplained symptoms gained more and more attention. This resulted—among others- in core changes in the diagnostic classification criteria of somatoform disorders. Against this background, the present “Perspective” will outline recent developments and findings in the area of medically unexplained somatic symptoms and somatoform disorders. Moreover, it will lay a special focus on evidence on specific negative psychological factors that may influence the course of unexplained somatic symptoms and disorders and the impairment caused by these symptoms.

## Introduction

Pain, gastrointestinal, cardiovascular, or other somatic symptoms which are not fully explained by a medical condition (medically unexplained symptoms), are very common both in the general population and in patients in health care (1–4). While most medically unexplained somatic symptoms are transient or do not cause impairment, in some cases they develop into chronic disabling complaints or full-blown somatoform disorders, which are associated with high health care utilization and severe impairment (5–8). In addition to the key role of impairing medically unexplained symptoms in somatoform disorders, there is evidence that persons with other mental disorders, such as depressive disorders, frequently suffer from medically unexplained symptoms and that medically unexplained symptoms may even negatively influence their course (9–15).

This “Perspective” will outline recent developments in the area of medically unexplained somatic symptoms and somatoform disorders from the perspective of the Author, with a special focus on psychological factors that may influence their course and the impairment caused by these symptoms.

## Somatoform disorders in the DSM and the ICD

Somatoform disorders are among the most frequent mental disorders, with prevalence rates estimated to be 5–6% in the general population (16). They were introduced as a diagnostic entity in the third version of the Diagnostic and Statistical Manual of Mental Disorders (DSM-III) (17) and were retained in the fourth version of the manual (DSM-IV) (18). In the DSM-IV, the prototype of somatoform disorders was *somatization disorder*, which was defined by at least eight medically unexplained somatic symptoms in four different organic systems persisting over several years and beginning before the age of 30 years. Similarly, the International Classification of Diseases, tenth version (ICD-10) (19), contained the diagnosis of *somatization disorder*, which was defined by at least six medically unexplained somatic symptoms in two different organic systems persisting over 2 years. However, prevalence rates for the somatization disorder were very low, i.e., ~0.4% in the general population and 0.5–6.0% in primary or secondary care (20–22). Moreover, the conceptualization was criticized with regard to utility and validity, since, among other things, evidence showed that many persons with multiple medically unexplained symptoms did not fulfill the strict criteria for somatization disorder despite their high impairment (23–25). In addition, somatization disorder was found to be associated with a strong recollection bias regarding symptoms (26). In general, the required dichotomization of bodily complaints into either “medically explained” or “medically unexplained” proved to be difficult even for specialists and brought about low interrater reliability {(27–29); but see (30) for an opposing perspective}. Occasionally, a transition of considering a symptom to be medically explained or not has occurred over time in both directions (4, 27, 31). The process of diagnosing was further complicated by patients whose complaints are related to a medical disease, but whose impairment exceeded the “expected” extent (32). Therefore, it was proposed that the dualistic distinction between “unexplained” and “medically explained symptoms” should be abandoned (33–36). This proposition was supported by a study in the general population, showing that most medically unexplained symptoms and medically explained symptoms resulted in comparable impairment and showed similar stability (37). The findings suggested that research should focus on the formulation and exploration of additional significant non-somatic classification criteria and factors that influence the impairment by medically unexplained symptoms, i.e., specifically on psychological factors. This should avoid shortcomings in diagnostic classification systems for somatoform disorders and consequently enable adequate management of impairing medically unexplained symptoms within the health care system. Taking into account the criticism outlined above, the DSM 5^th^ edition (DSM-5) (38) and the ICD 11^th^ edition (ICD-11) revised the former sections of somatoform disorders. In the DSM-5, some of the former somatoform disorders were replaced with the new diagnosis of *somatic symptom disorder* (300.82). For this diagnosis, the former differentiation between medically unexplained and explained somatic symptoms was abandoned, such that medically explained symptoms also counted for the core classification criterion of impairing symptoms. In addition, psychological classification criteria (criterion B) were included (see section 3 below). Similarly, the ICD-11 (39) introduced the new classification *bodily distress disorder* which is characterized by persistent and distressing somatic symptoms (including medically explained symptoms) which draw excessive attention.

## Psychological factors in medically unexplained symptoms and somatoform disorders

Since persistent medically unexplained somatic symptoms and somatoform disorders bring about high costs for health care systems and are among the leading causes of disability (8), it is highly relevant to investigate psychological factors that characterize and influence these symptoms and disorders. The intensity of and impairment by medically unexplained symptoms, i.e., their interference with daily life, as well as health care utilization, are seen as core outcome criteria in the treatment of persons suffering from somatoform disorders (40). Therefore, the investigation of psychological factors that influence these criteria is of major importance in order to improve the diagnosis and treatment of affected persons. Furthermore, the investigation of mechanisms underlying the associations between medically unexplained symptoms and their perceived intensity and impairment is of high interest for the provision of appropriate and timely intervention strategies.

Evidence suggested that in addition to more unspecific factors such as early childhood trauma or insecure attachment (41), specific *negative psychological factors* such as catastrophizing, negative affectivity, rumination, avoidance, health anxiety, or a negative physical self-concept have a substantial influence on the transition from unproblematic medically unexplained somatic symptoms to severely impairing complaints and somatoform disorders. Individuals may differ in the extent to which negative psychological factors occur. Evidence suggested that persons with chronic and disabling medically unexplained symptoms and somatoform disorders show more negative psychological factors than do persons without such symptoms, and that negative psychological factors strongly influence the impairment and illness behavior of persons with chronic medically unexplained symptoms as well as the stability of these symptoms (42–44). Individuals with more negative psychological factors may perceive medically unexplained symptoms as more threatening and may consequently show a higher cognitive, emotional, and behavioral awareness of these symptoms. For instance, a recent study in the general population by Toussaint et al. (45) found that persons who suffered from somatic symptoms and a high degree of psychological symptoms related to the somatic symptoms (i.e., persons who fulfilled the criteria for a somatic symptom disorder) reported to spend eight times more time a day dedicated to their somatic symptoms (4 h/day) in comparison to persons with less somatic symptoms and way lower psychological symptoms (half an hour/day). This process may, in turn, lead to increased negative bodily sensations, resulting in a higher intensity of and impairment by medically unexplained symptoms (43, 46–49). Indeed, Toussaint et al. (45) found that the psychological symptoms were the strongest (cross-sectional statistical) predictor for the self-rated health status in their general population sample.

The topic of psychological factors also bears relevance with regard to the classification of impairing medically unexplained symptoms and somatoform disorders. To justify the classification of somatoform disorders as a DSM or ICD section F/mental disorders diagnosis (18, 19, 38), positive psychological classification criteria were required (25). A study in the general population evaluated specific negative psychological factors that could be used as classification criteria for impairing somatic/somatoform syndromes requiring health care {e.g. (42, 44)}. Specifically, it aimed to determine the relevance of these negative psychological factors with regard to impairment by (medically unexplained) somatic symptoms and health care utilization due to these symptoms. These criteria should help to identify those people who need health care, as compared to people who are able to cope with their symptoms themselves, without health care. Moreover, the criteria should identify those patients who are seriously impaired by the symptoms, in contrast to those who have some symptoms but do not feel impaired. The study authors found several negative psychological factors that might influence whether persons with somatic symptoms require health care and/or feel impaired by their symptoms: (1) ruminations about somatic complaints and worrying about health and illness; (2) catastrophizing of bodily sensations; (3) somatic illness attributions despite contradictory medical information; (4) a self-concept of bodily weakness; (5) low symptom tolerance and immediate need for medical help when symptoms occur; (6) avoidance of physical activity that could cause sweating or heart rate acceleration; (7) disuse of body parts because of complaints; (8) feelings of desperation because of symptoms and negative affectivity. Further, longitudinal analyses showed that persons fulfilling the negative psychological factors reassurance seeking, body checking, catastrophizing of physical sensations, avoidance of physical activities, a self-concept of bodily weakness„ and negative affectivity had a two to ten higher odds ratio for suffering from a somatoform disorder 1–4 years later, with up to 90% correct predictions for the overall model (42). Other studies used the comparison between different alternative classification proposals {e.g., bodily distress disorder introduced by Fink et al. (50), polysymptomatic disorder introduced by Rief et al. (51)} to determine the possible value of specific psychological classification criteria (51, 52). They found that the inclusion of psychological and behavioral criteria increased the concurrent validity of the proposals and partly also the predictive validity.

Based on the evidence outlined above, the DSM-5 (38) and the ICD-11 (39) revised their former sections of somatoform disorders, and included specific psychological criteria, i.e., health anxiety, catastrophizing, or high time or energy devoted to the preoccupation with somatic symptoms in the DSM-5, and excessive attention that can not be alleviated by clinical examinations and reassurance of innocuousness in the ICD-11. Nevertheless, the described findings suggested that, although the validity of the diagnoses was improved by the inclusion of psychological classification criteria {for a recent scoping review on evidence on somatic symptom disorder please see (41)}, there were some shortcomings with regard to the limited number of considered negative psychological factors. For instance, it would be advisable to widen somatic symptom disorder's psychological criterion (criterion B) through the inclusion of a self-concept of bodily weakness and negative affectivity, and also to specify the existing criteria with regard to rumination and avoidance (42, 44). Similarly, the bodily distress disorder may benefit from including a broader range of psychological criteria and/or further specification of “excessive attention” (i.e., with regard to behavioral, emotional, and cognitive indications). In this regard, the study of Toussaint et al. mentioned above (45) took an important first step in shedding light on the “excessiveness” in terms of daily time dedicated to somatic symptoms. Further refinement of the diagnostic criteria may help to even better meet the requirements regarding validity and consequently the needs of patients with mainly medically unexplained symptoms, their treating clinicians, and researchers.

## Psychological factors in the daily lives of persons suffering from medically unexplained symptoms

Despite the dynamic trajectories and volatility of medically unexplained symptoms (4, 37, 53–56), most studies investigating medically unexplained symptoms and negative psychological factors used rather static data, i.e., questionnaires or data from only one time point, or assessed persons in the laboratory, i.e., in a rather artificial setting far removed from their daily life. While these studies provided valuable insights into how to establish the differential relationships between medically unexplained symptoms, negative psychological factors, and impairment, they were unable to capture dynamic associations and mechanisms, and their results may not be generalizable to individuals' daily life. To elucidate the dynamic associations between negative psychological factors and the intensity of and impairment by medically unexplained symptoms, a micro-longitudinal design using ecological momentary assessment (EMA) may represent the best choice. An EMA approach has the potential to provide insight into the occurrence of negative psychological factors and specific reactions as they actually occur in everyday life (57–61). Moreover, such an approach avoids the limitations of cross-sectional or longer-term longitudinal designs {such as the inability to test causal relationships, low temporal resolution, memory biases, and losses to follow-up assessments (55)}, and of experimental approaches (such as the lack of generalizability of observed relationships).

Only a handful of studies have investigated associations between single negative psychological factors or stress and impairment by somatic symptoms using ambulatory assessment designs (48, 53, 56, 62–65). The respective findings suggest negative influences of negative psychological factors and stress on daily somatic symptoms in healthy students or persons suffering from functional somatic syndromes/medically unexplained symptoms. However, these studies were limited both in generalizability and ecological validity, as they mainly investigated small groups, focused on pain and single psychological factors, had very short assessment periods, or included a low number of assessments per day. Two studies investigated the relevance of several specific negative psychological factors in the daily life of women suffering from medically unexplained symptoms using an EMA design with several assessments per day over a period of 14 days (66, 67). They focused exclusively on women due to the female preponderance regarding somatoform disorders/somatic symptom disorder and depressive disorders (8, 68) and given the sex-specific differences in biological responses to stress (69–71).

The first study investigated the everyday life occurrence of negative psychological factors in women suffering from chronic medically unexplained symptoms in the form of widespread pain (fibromyalgia syndrome) (66). In addition, the predictive value of negative psychological factors concerning the intensity of and impairment by the pain was investigated. In this study, ambulatory data were assessed over 14 consecutive days with six daily assessments via an iPod. Twenty-eight women suffering from chronic widespread pain estimated the strength of three negative psychological factors (somatic illness beliefs, health anxiety, time/energy devoted to pain or health concerns) and the intensity of momentary pain. The results showed that, on average, negative psychological factors occurred three to four times per day and had a mild to moderate severity. Interestingly, they were both concurrently and prospectively associated with momentary pain intensity and subjective impairment by pain. Negative psychological factors and pain medication explained 20% of the variance in pain intensity and 28% of the variance in subjective impairment.

The second study also included biological measures, as a major aspect of the negative consequences of negative psychological factors is their potential to elicit biological stress responses (67, 72, 73). These responses are coordinated by a complex system encompassing the hypothalamic-pituitary-adrenal (HPA) axis and the autonomic nervous system (74–78), and may in turn also influence the intensity of and impairment by medically unexplained symptoms (65). Previous studies showed that the activity of these systems was differentially affected in persons with somatic symptom disorder and persons with depressive disorders. While the activity of the HPA axis is assumed to be reduced in individuals with impairing medically unexplained symptoms (79–81), HPA axis hyperactivity is apparent in persons with depressive disorders (82, 83). A recent meta-analysis even found that the higher the cortisol levels in persons with depressive disorders at the start of psychological therapy, the worse the outcome at the end of treatment (84). In the EMA study, 29 women with somatic symptom disorder (based on medically unexplained somatic symptoms) and 29 women with depressive disorders participated. In this study, intensity of and impairment by somatic symptoms, negative psychological factors, and stress biomarkers (cortisol and alpha-amylase) were assessed five times per day over 14 consecutive days using an electronic device and saliva samples. The results showed that the more negative psychological factors were present, the higher were the concurrent and time-lagged intensity of and impairment by somatic symptoms in women with somatic symptom disorder and with depressive disorders. In women with depressive disorders, negative psychological factors were associated with higher levels of salivary cortisol. In contrast, they were associated with lower levels in women with somatic symptom disorder. In women with somatic symptom disorder, lower cortisol levels were associated with higher intensity at the next measurement time point, i.e., 3–4 h later, emphasizing the utility of stress-reducing interventions in this group (67).

The two EMA studies impressively demonstrated the strong immediate and delayed impact of specific negative psychological factors on the intensity of and impairment by somatic symptoms in the daily life of affected persons with different disorders. Thus, negative psychological factors may be considered as transdiagnostic factors in the development and treatment of impairing (medically unexplained) somatic symptoms. With the unique combination of subjective and biological measures the second study found support for the possible mediating role of the HPA axis in the association between negative psychological factors and the suffering from somatic symptoms. These results are highly relevant, as they can inform the development of new treatment strategies which use ecological momentary intervention approaches focusing on negative psychological factors in persons suffering from impairing somatic symptoms (85). Since the two EMA studies only included women without any medical condition that may affect endocrine or autonomic functioning (because of the investigated biological markers), the generalizability of the findings to persons with such a medical condition remains unclear. Since studies showed that specific negative psychological factors may aggravate somatic complaints accompanying medical illnesses to an extent that cannot be fully explained by the underlying illness (86–89), the findings of the EMA studies may bear some relevance for persons suffering from a medical condition. However, the inclusion of medical conditions may have changed the characteristics of the investigated group and the strength of the presented psychological factors, since a study suggested that the diagnosis of somatic symptom disorder becomes less strict when medically explained somatic symptoms are included (90). With the lack of clear criteria for the fulfillment of the B criteria for somatic symptom disorder in the presence of medical conditions, the diagnosis may become less reliable and may lose validity. Future studies should shed light on this important issue.

## Discussion

The presented evidence showed the relevance of specific negative psychological factors for the conceptualization, the diagnosis, and the treatment of medically (un)explained symptoms and various diagnostic entities in which these symptoms are pathognomonic, and showed recent developments in this regard.

The findings underlined the importance to consider negative psychological factors in the context of medically unexplained symptoms, as these factors may have the potential to explain why medically unexplained somatic symptoms cause so much impairment without a (known) underlying medical disease. Indeed, the evidence outlined confirmed the high relevance of specific negative psychological factors for the concurrent and predictive intensity of and impairment by medically unexplained symptoms in the general population. It showed that specific negative psychological factors contributed to the maintenance of multiple impairing medically unexplained symptoms over several years, as well as to the direct impairment by somatic symptoms in the daily lives of affected persons. A recent EMA study even suggested that these specific negative psychological factors were transdiagnostic, since they were equally relevant for the impairment by somatic symptoms in women with depressive disorders as they were in women with somatic symptom disorder.

Moreover, the presented findings suggest that for persons suffering from medially unexplained somatic symptoms, the current classification criteria for somatic symptom disorder and bodily distress disorder might be further improved by including additional psychological classification criteria (e.g., reassurance seeking, body checking, a self-concept of bodily weakness, avoidance behavior, and negative affectivity) or by the use of these criteria/factors to specify the current psychological criteria. This could improve the early detection and timely treatment of persons at risk for a chronic course of somatoform disorders/somatic symptom disorder/bodily distress disorder. However, it is important to note that while the suggestions for additional psychological classification criteria is based on a broad evidence [see above and (41)], there is no consensus on the exact set of psychological criteria that may be relevant for a diagnosis in the field of somatoform disorders. Moreover, the relevance of specific criteria may vary between cultures [e.g., (90, 91)].

Despite the intriguing relevance of psychological classification criteria, there may also be cases where psychological classification criteria should not be mandatory for a diagnosis. As Burton et al. (92) suggest in their proposition of the category *functional somatic disorders*, there may be need for a diagnosis that captures persons suffering from persistent impairing functional somatic symptoms or syndromes (e.g., fibromyalgia or irritable bowel syndrome), who may or may not fulfill additional psychological criteria {for a recent review on functional somatic syndromes also see (93)}.

The findings of the studies using an EMA design provided further scientific groundwork for treatments of persons suffering from chronic medically unexplained symptoms. They supported the rationale of treatment approaches focusing on cognitive-behavioral factors in general (94), as well as approaches considering negative affectivity and emotion regulation (95, 96) and avoidance (97) in particular. Furthermore, they can inform the development of new treatment strategies which use ecological momentary intervention approaches to reduce negative psychological factors in persons suffering from impairing somatic symptoms (85). Future studies should follow this promising avenue.

## Data availability statement

The original contributions presented in the study are included in the article/supplementary material, further inquiries can be directed to the corresponding author.

## Author contributions

The author confirms being the sole contributor of this work and has approved it for publication.

## Conflict of interest

The author declares that the research was conducted in the absence of any commercial or financial relationships that could be construed as a potential conflict of interest.

## Publisher's note

All claims expressed in this article are solely those of the authors and do not necessarily represent those of their affiliated organizations, or those of the publisher, the editors and the reviewers. Any product that may be evaluated in this article, or claim that may be made by its manufacturer, is not guaranteed or endorsed by the publisher.
